# Role of *sfk1* Gene in the Filamentous Fungus *Penicillium roqueforti*

**DOI:** 10.3389/fmicb.2017.02424

**Published:** 2017-12-06

**Authors:** Claudia Torrent, Carlos Gil-Durán, Juan F. Rojas-Aedo, Exequiel Medina, Inmaculada Vaca, Paulo Castro, Ramón O. García-Rico, Milena Cotoras, Leonora Mendoza, Gloria Levicán, Renato Chávez

**Affiliations:** ^1^Departamento de Biología, Facultad de Química y Biología, Universidad de Santiago de Chile, Santiago, Chile; ^2^Departamento de Química, Facultad de Ciencias, Universidad de Chile, Santiago, Chile; ^3^Departamento de Química de los Materiales, Facultad de Química y Biología, Universidad de Santiago de Chile, Santiago, Chile; ^4^GIMBIO Group, Department of Microbiology, Faculty of Basic Sciences, Universidad de Pamplona, Pamplona, Colombia

**Keywords:** *Penicillium roqueforti*, suppressor of four kinase, RNA-mediated gene silencing, phenotypic changes, secondary metabolites

## Abstract

The *sfk1* (suppressor of four kinase) gene has been mainly studied in *Saccharomyces cerevisiae*, where it was shown to be involved in growth and thermal stress resistance. This gene is widely conserved within the phylum *Ascomycota*. Despite this, to date *sfk1* has not been studied in any filamentous fungus. Previously, we found that the orthologous of *sfk1* was differentially expressed in a strain of *Penicillium roqueforti* with an altered phenotype. In this work, we have performed a functional characterization of this gene by using RNAi-silencing technology. The silencing of *sfk1* in *P. roqueforti* resulted in decreased apical growth and the promotion of conidial germination, but interesting, it had no effect on conidiation. In addition, the attenuation of the *sfk1* expression sensitized the fungus to osmotic stress, but not to thermal stress. RNA-mediated gene-silencing of *sfk1* also affected cell wall integrity in the fungus. Finally, the silencing of *sfk1* depleted the production of the main secondary metabolites of *P. roqueforti*, namely roquefortine C, andrastin A, and mycophenolic acid. To the best of our knowledge this is the first study of the *sfk1* gene in filamentous fungi.

## Introduction

Fungi use a complex network of signal transduction pathways to sense abiotic and biotic signals from the environment (Park et al., 2010). Therefore, signal transduction pathways have pivotal importance in fungi. Currently, signal transduction pathways in model fungi such as *Saccharomyces cerevisiae* and *Aspergillus* spp. have been widely studied. Conversely, these pathways have been scarcely studied in other species such as the filamentous fungus *Penicillium roqueforti. P. roqueforti* is important to the food industry, particularly in the production of several kind of ripened blue-veined cheeses (Fernández-Bodega et al., 2009). In addition, this fungus produces several interesting secondary metabolites (García-Estrada and Martín, 2016). Despite this, cellular processes in *P. roqueforti* have been poorly studied.

In *P. roqueforti*, the most studied signal transduction pathway corresponds to the heterotrimeric G proteins, particularly the α-subunit from the subgroup I (Gαi). By overexpressing a dominant active Gαi subunit, it has been demonstrated that this protein influences several processes in the fungus. Thus, this protein negatively affects growth, sporulation, and thermal and osmotic stress resistance in *P. roqueforti* (García-Rico et al., 2007, 2008a, 2009). Furthermore, Gαi stimulates germination and increases the production of the secondary metabolite roquefortine C (García-Rico et al., 2009).

Gαi signaling produces pleiotropic effects on fungal cells. Therefore, Gαi could be affecting several other regulators. In *P. roqueforti*, Gαi signaling negatively affects the expression of *pcz1*, a gene encoding for a Zn(II)_2_Cys_6_ protein (Gil-Durán et al., 2015). Interestingly, *pcz1* showed a positive effect on apical extension and conidiation, whereas it had negative effect on conidial germination (Gil-Durán et al., 2015).

To date, few molecular determinants involved in the control of cellular processes in *P. roqueforti* have been described. With the aim of unveiling genes from *P. roqueforti* that could be related with Gαi signaling, in a previous work we performed suppression subtractive hybridization (SSH) experiments (Gil-Durán et al., 2015). From these experiments, several cDNA sequences differentially expressed were obtained, including a full cDNA corresponding to the orthologous of the *sfk1* (suppressor of four kinase) gene from *S. cerevisiae*.

The *sfk1* gene was originally discovered in *S. cerevisiae*, and encodes for a transmembrane protein (Sfk1) located at the plasma membrane, whose specific function is the proper localization of a phosphatidylinositol 4-kinase named Stt4 to the membrane (Audhya and Emr, 2002). Stt4 is an enzyme involved in the phosphoinositide second messengers pathway that regulate several cellular processes in the yeast (Audhya et al., 2000), and to fulfill its role, Stt4 must be tethered to the plasma membrane by Sfk1 (Voelker, 2005).

A preliminary inspection of GenBank database records indicates that the orthologs of Sfk1 are widely conserved within the phylum *Ascomycota* (unpublished data), but strikingly, to date this gene has not been studied in filamentous fungi. Therefore, we decide to characterize this gene in *P. roqueforti*.

## Materials and Methods

### Fungal Strain and Culture Media

The wild-type strain *P. roqueforti* CECT 2905 (ATCC 10110) was used in this work. The strain was routinely kept on Power medium agar (Fierro et al., 1996). Other media used in this work were Czapek minimal medium, YES agar (Rojas-Aedo et al., 2017) and CM liquid medium (Gil-Durán et al., 2015).

### Construction of Plasmid pSfk1-RNAi for *sfk1* Silencing and Transformation of *P. roqueforti* CECT 2905

To generate the *sfk1* knockdown construct, plasmid pJL43-RNAi (Ullán et al., 2008) was used. This plasmid has two promoters in opposite directions, and an *Nco*I site between the promoters (Ullán et al., 2008).

A 450-bp fragment of the *sfk1* gene was amplified with primers RiSfkF (5′- AGACTCCCATGGCCACTGGACTATGATCG -3′) and RiSfkR (5′- AGACTCCCATGGAACGACAATGAACGAGGA -3′). Both primers contain an *Nco*I site (underlined). The amplified product was digested with *Nco*I and ligated into pJL43-RNAi (also previously digested with *Nco*I and dephosphorylated), giving rise to plasmid pSfk1-RNAi. The pSfk1-RNAi plasmid was confirmed by sequencing (data not shown) and used to transform *P. roqueforti* CECT 2905 according to the protocol described by Gil-Durán et al. (2015). In addition, a control strain that contains the empty plasmid pJL43-RNAi was obtained.

### Nucleic Acids Extractions and RT-PCR Assays

DNA and total RNA from *P. roqueforti* were extracted as described by Gil-Durán et al. (2015). RT-PCR experiments were performed as described by García-Rico et al. (2017). Briefly, total RNA extracted was treated with RNase-free DNase I (Roche, Germany) and then quantified using a μDrop plate in a MultiSkan GO quantification system (Thermo Scientific, Germany). To synthesize *sfk1* cDNA, RevertAid Reverse Transcriptase (Thermo Scientific, Germany) was used according the manufacturer’ instructions. Reactions contained 200 ng of treated RNA and 10 pmol of primers RiSfkF and RiSfkR. Amplification of β-tubulin cDNA (internal control) was performed as described by García-Rico et al. (2017). The products of the reactions were resolved by electrophoresis and quantified by densitometry analysis using the “myImageAnalysis” software (Thermo Scientific, Germany). Values were expressed as percentage of the ratio between *sfk1*/β-tubulin.

### Phylogenetic Analysis

The deduced sequence of Sfk1 protein from *P. roqueforti* CECT 2905 was used to find similar sequences using BlastP. Sequences obtained were used to perform phylogenetic analysis. Briefly, sequences were aligned by Clustal Omega and the phylogenetic trees were reconstructed using the Neighbor-joining method with the MEGA version 7 program (Kumar et al., 2016). Evolutionary distances data were calculated using the Poisson correction model. The support of internal nodes was performed by bootstrap analyses with 1,000 replications.

### Phenotypic Characterization, Analysis of Thermal and Hypertonic Stress Resistance, and Cell Wall Integrity Assay

The apical extension rates, the production of conidia, and the measurements of conidial germination were done exactly as described by Gil-Durán et al. (2015). The thermal and hypertonic stress analyses were performed as described by García-Rico et al. (2017).

The cell wall integrity assays were performed using calcofluor white as described by Mendoza et al. (2016). Briefly, conidia from *P. roqueforti* at a final concentration of 1 × 10^4^ conidia/mL were inoculated in 24-well plates (lined with 12-mm glass coverslips) containing 1 mL of CM medium. Cultures were incubated at 28°C for 15 h until the germination of conidia. After this time, the medium was removed and glass coverslips with *P. roqueforti* hyphae adhered were washed three times with 0.9% NaCl, and stained with calcofluor white during 10 min. Then the glass coverslips containing hyphae were washed again and mounted on slides. The fluorescence of *P. roquerfoti* hyphae stained with calcofluor white was observed under a fluorescence microscope with Blue/Violet (110033V2) filter. The fluorescence intensity was quantified using the program “myImageAnalysis” v2.0 (Thermo Scientific, Germany). For this purpose, the fluorescence intensity of hyphae from three randomly-chosen fields of each sample was recorded. The average of the fluorescence intensities recorded from the wild-type strain was set as 100% of fluorescence. The rest of the samples were normalized with respect to the wild-type strain. As control of cell wall damage, germinated conidia from the wild-type strain were treated with Lysing Enzymes from *Trichoderma harzianum* (20 mg/mL) during 2 h at 28°C, and stained with calcofluor white as above.

### Extraction of Secondary Metabolites and HPLC Analyses

The production, extraction and HPLC analyses of mycophenolic acid and andrastin A from *P. roqueforti* CECT 2905 have been described elsewhere (Del-Cid et al., 2016; Rojas-Aedo et al., 2017). In the case of roquefortine C, the strains were grown on YES agar for 15 days at 28°C. The mycelia were collected, suspended in a chloroform-methanol (2:1) mixture, and then submitted to sonication during 30 min. The mixture was filtered through nylon membranes (mesh 30 μm). The extracted mycelia were kept for further dry-weight determination (see below), whereas the filtrated was evaporated to dryness in a rotary evaporator. Previous to HPLC analysis, the dried filtrated was suspended in 500 μl of methanol (HPLC grade) and briefly centrifuged. Twenty μl of this suspension were submitted to HPLC analysis. HPLC analysis was performed using a Waters 1525 HPLC equipment (Waters, Ireland) with a 4.6 mm × 250 mm (5 μm) SunFire C18 reverse phase column. The column was held at 35°C. The mobile phase was water with 0.02% TFA (solvent A) and acetonitrile with 0.02% TFA (solvent B). The gradient program was as follows: 15% B to 47% B linear over 15 min, 47% B to 100% B over 1 min, and isocratic 5 min. The flow rate used was 1.2 mL/min. For the detection and quantification of the compound, pure roquefortine C (Santa Cruz Biotechnology) was used as standard. The detection of roquefortine C was performed at 254 nm. Under the conditions described, roquefortine C showed a retention time of 10.9 min. Roquefortine C production was normalized by dry weight of fungal mycelia. For this purpose, the mycelium from each sample was dried as described by García-Rico et al. (2009).

### Nucleotide Sequence Accession Number

The nucleotide sequence of the *sfk1* gene described in this work has been deposited in the GenBank database under accession number MF614690.

## Results

### Analysis of *sfk1* Sequence

In a previous work, we performed SSH experiments to looking for genes from *P. roqueforti* CECT 2905 that are regulated by Gαi (Gil-Durán et al., 2015). Among the sequences obtained from these experiments, a full cDNA matching with hypothetical *sfk1* genes from ascomycetous fungi (mainly *Penicillium* species) was obtained. This cDNA sequence is 914 nt- long, excluding the poly-A tail. By using genomic DNA from *P. roqueforti* CECT 2905 and suitable primers (designed from the cDNA sequence), we amplified the *sfk*1 gene by PCR. The gene was sequenced and compared with the cDNA sequence, revealing the presence of four introns (data not shown). The *sfk1* gene from strain CECT 2905 was also submitted to BlastN analysis against the genome of *P. roqueforti* FM164 (Cheeseman et al., 2014). We found that *sfk1* gene from *P. roqueforti* CECT 2905 exactly matches (100% identity, 100% coverage, and no gaps) with the *sfk1* gene from strain FM164, located at contig ProqFM164S01-10 in the genome sequence of this strain (data not shown).

**FIGURE 1:**
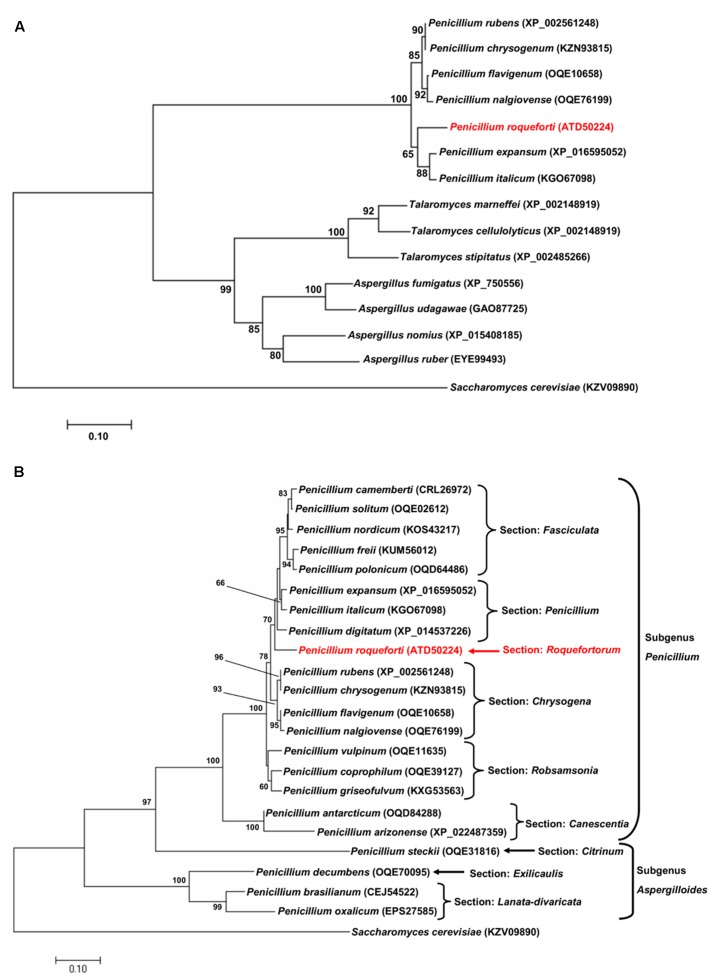
Evolutionary relationships of Sfk1 from *Penicillium roqueforti* CECT 2905 and its orthologous proteins. **(A)** Evolutionary tree of Sfk1 proteins from species from the genera *Penicillium*, *Aspergillus*, and *Talaromyces*. The sequence from *P. roqueforti* is highlighted in red. In parenthesis, the accession number of each sequence is indicated. Sfk1 from *Saccharomyces cerevisiae* was used as outgroup. Bootstrap values (50% or higher) are shown in each node. **(B)** Evolutionary tree of all Sfk1 proteins from the genus *Penicillium* retrieved from BlastP analysis. The sequences are present in *Penicillium* species assigned to different sections within the subgenera *Penicillium* or *Aspergilloides*. The sequence from *P. roqueforti* is highlighted in red. In parenthesis, the accession number of each protein sequence is indicated. Sfk1 from *S. cerevisiae* was used as outgroup. Bootstrap values (50% or higher) are shown in each node.

The *sfk1* gene from *P. roqueforti* CECT 2905 encodes a protein of 235 amino acids. BlastP analysis of the deduced protein revealed the presence of orthologs in a wide range of species of filamentous fungi from the phylum *Ascomycota*, mainly from the genera *Penicillium*, *Aspergillus*, and *Talaromyces*. Using several of these sequences, a phylogenetic analysis was performed (**Figure 1A**). This analysis revealed that Sfk1 sequences are clustered in agreement with the expected evolutionary relationship among these genera. On the other hand, using all Sfk1 sequences from *Penicillium* species retrieved by BlastP (22 sequences including Sfk1 from *P. roqueforti*), a second phylogenetic analysis was performed (**Figure 1B**). Eighteen sequences (including Sfk1 from *P. roqueforti*) belong to species from the subgenus *Penicillium*, whereas five sequences belong to species from the subgenus *Aspergilloides* (**Figure 1B**). In this case, the Sfk1 sequences also are clustered in agreement with the expected evolutionary relationship among these *Penicillium* species (**Figure 1B**).

Finally, and according to bioinformatics predictions (TMHMM Server v. 2.0 and TMpred at Expasy), the Sfk1 protein from *P. roqueforti* is a transmembrane protein. It contains six putative transmembrane helix domains, with its amino and carboxyl ends inside oriented (data not shown).

### RNA-Mediated Gene-Silencing of *sfk1*

**FIGURE 2:**
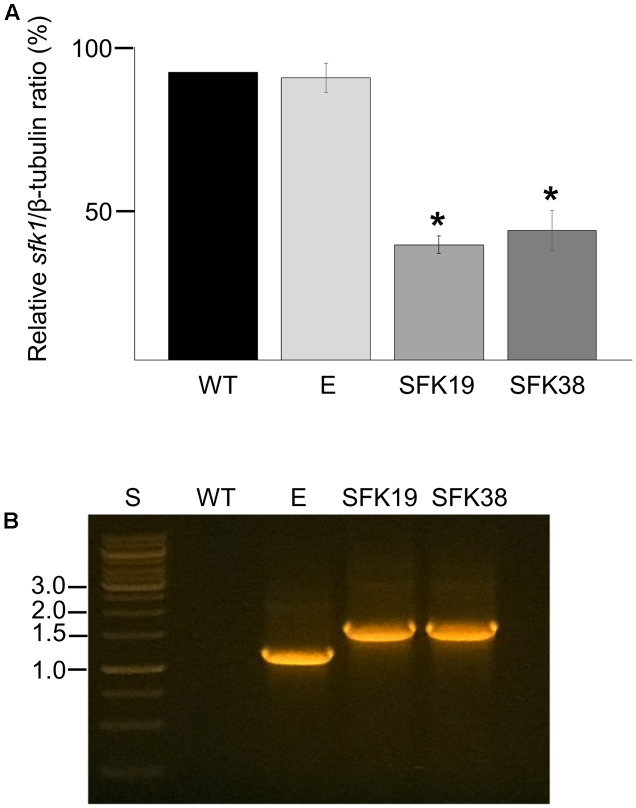
Gene silencing of *sfk1* in *P. roqueforti*. **(A)** Transcript levels of *sfk1* and β-tubulin in *P. roqueforti* strains were quantified by densitometry analysis. The *sfk1*/β-tubulin ratio of the wild-type (WT) strain was normalized to 100%, whereas the *sfk1*/β-tubulin ratios of the transformants were normalized with respect to the *sfk1*/β-tubulin ratio of the wild-type strain. The symbol ^∗^ indicates that differences were statistically significant (*p* < 0.05, Student’s *t*-test). E: control strain containing empty pJL43-RNAi. **(B)** PCR assay demonstrating integration of the full silencing cassette in transformants SFK19 and SFK38. PCR products were subjected to electrophoresis in agarose gels. Lane WT: wild-type strain *P. roqueforti* CECT 2905; lane E: *P. roqueforti* containing empty pJL43-RNAi vector; lane S: standard O’GeneRuler 1 kb DNA Ladder, ready-to-use (Thermo Scientific). Relevant sizes are shown at left.

RNA-mediated gene silencing was used to perform the functional characterization of *sfk1* in *P. roqueforti* CECT 2905. The fungus was transformed with pSfk1-RNAi, and 57 phleomycin-resistant transformants were obtained. Fifteen transformants were randomly selected and subjected to RT-PCR (see section “Materials and Methods”). Two of these transformants (hereafter SFK19 and SFK38) showed a reduction of *sfk1* transcripts of approximately 55% compared to the wild-type strain (**Figure 2A**). In addition, the presence of silencing cassette in these transformants was also confirmed by PCR (**Figure 2B**). Taken together, all these data indicate the successful silencing of *sfk1* in transformants SFK19 and SFK38.

### The Knockdown of *sfk1* Decreases Growth Rate and Increases Conidial Germination, But Does Not Affect Conidiation in *P. roqueforti*

**FIGURE 3:**
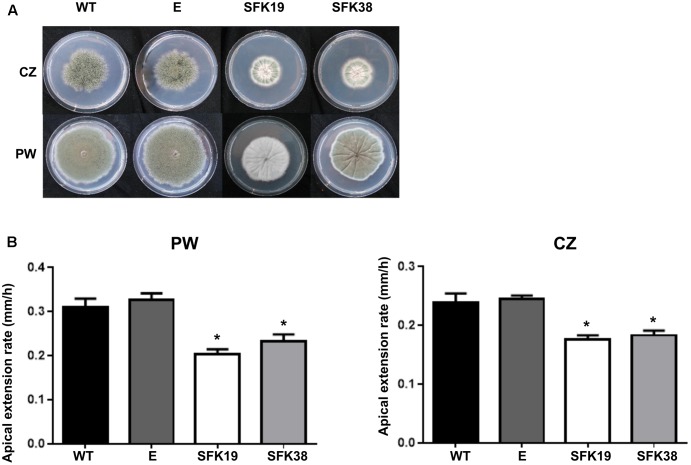
Effect of RNA-mediated gene-silencing of *sfk1* on growth of *P. roqueforti*. **(A)** Phenotypes of *P. roqueforti* CECT 2905 (WT) and transformants SFK19 and SFK38. The colonies were grown for 7 days at 28°C in poor Czapek (CZ) or rich Power (PW) media. Note the reduction in growth of strains SFK19 and SFK38 as compared to the wild-type strain, especially in Czapek medium. *P. roqueforti* containing empty pJL43-RNAi vector (E) is included as control. This strain was undistinguishable from the wild-type strain. **(B)** Comparison of apical extension rates (mm/h) of *P. roqueforti* strains on Power (PW) and Czapek (CZ) media. Error bars represent standard deviation of three replicas from three different experiments. The symbol ^∗^ indicates that differences were statistically significant (*p* < 0.05, Student’s *t*-test).

Since the deletion of *sfk1* produces changes in the growth pattern of *S. cerevisiae* (Jin et al., 2008), we analyzed the effect of the RNA-mediated silencing of *sfk1* on growth in *P. roqueforti*. **Figure 3A** shows that the colonies of the transformants SFK19 and SFK38 grown on Czapek minimal medium or Power rich medium were smaller than the wild-type strain, suggesting that the knockdown of *sfk1* altered growth of *P. roqueforti*. **Figure 3B** shows the apical extension rates of transformants SFK19 and SFK38 on the same media. As observed, and independently from the medium used, the attenuation of *sfk1* altered normal growth of the fungus. On Czapek medium, the apical growth rate of strains SFK19 and SFK38 ranged between 0.170 and 0.185 mm/h, versus 0.240 mm/h in the wild-type strain, whereas on Power medium, the apical growth rate of strains SFK19 and SFK38 ranged between 0.20 and 0.22 mm/h, versus 0.31 mm/h in the wild-type strain (**Figure 3B**). These results indicate that on Czapek medium, the transformants grew at a rate between 72 and 77% of wild-type strain levels. Similarly, on Power medium the transformants grew at a rate between 64 and 75% of wild-type strain levels. Therefore, we conclude that the knockdown of *sfk1* in *P. roqueforti* caused a reduction in the apical extension rate.

**FIGURE 4:**
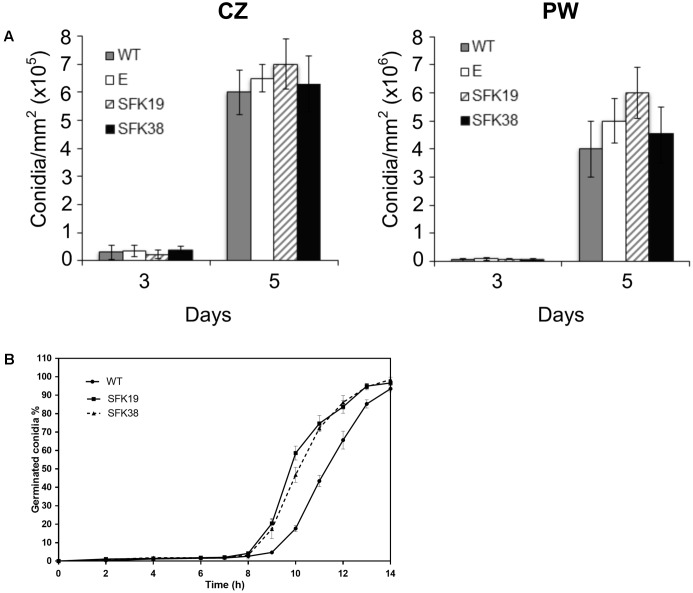
Effect of RNA-mediated gene-silencing of *sfk1* on conidiation and conidial germination of *P. roqueforti*. **(A)** Conidial production by *P. roqueforti* CECT 2905 and transformants SFK19 and SFK38.5 × 10^4^ conidia were seeded in Petri dishes containing Czapek (CZ) or Power (PW) media. Dishes were incubated at 28°C for 3 or 5 days, and the conidia produced were collected and counted as described by Gil-Durán et al. (2015). Values are expressed as conidia/mm^2^ of surface. A strain containing empty pJL43-RNAi vector (E) was used as a control. Error bars represent the standard deviation of three replicas from three independent experiments. The differences in conidial production of strains SFK19 and SFK38 were statistically undistinguishable (*p* < 0.05 using Student’s *t*-test) as compared to the wild-type and control strains. **(B)** Germination kinetics of *P. roqueforti* CECT 2905 (WT) and transformants SFK19 and SFK38. Conidia from each strain were inoculated in CM liquid media and incubated at 28°C. At regular intervals, samples were taken, and the number of germinated and non-germinated conidia was counted under the microscope in 10 randomly-chosen fields. Data were expressed as the percentage of germinated conidia vs. hours of incubation. Error bars represent the standard deviation of three replicates in three independent experiments. No differences were observed between the wild-type strain and *P. roqueforti* containing empty pJL43-RNAi vector (data not shown).

We also tested conidia production in all the strains. Interestingly, we found that the RNA-mediated silencing of *sfk1* does not affect conidiation (**Figure 4A**). However, the knockdown of *sfk1* affected conidial germination. **Figure 4B** shows that all the strains follow a sigmoidal pattern of conidial germination, but in transformants SFK19 and SFK38, this process was earlier (around 1 h) compared with the wild-type strain. For example, at 9 h, around 20% of conidia from transformants have already germinated, whereas the wild-type strain reaches the same percentage of germination at 10 h. Similar differences are observed along all the exponential phase of germination (**Figure 4B**).

### The Knockdown of *sfk1* Decreases Hypertonic Stress Resistance But Does Not Affect Heat Shock Resistance in *P. roqueforti*

**FIGURE 5:**
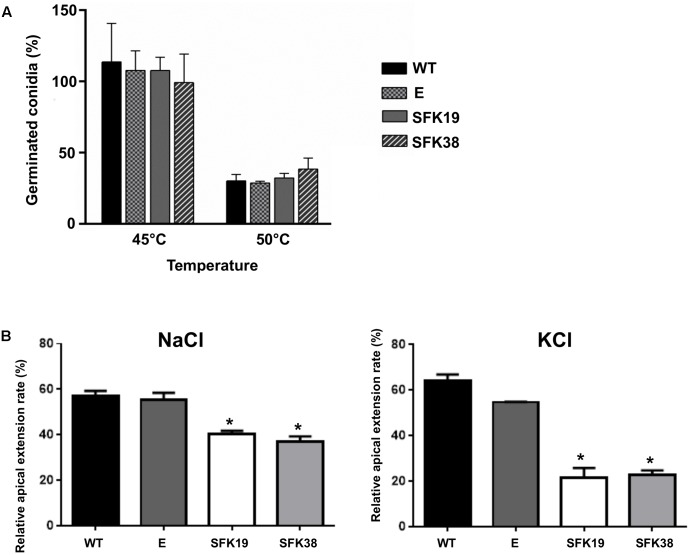
Analysis of thermal hypertonic stress resistance of *P. roqueforti* CECT 2905 and transformants SFK19 and SFK38. **(A)** Percentage of viable conidia after 1 h of heat shock treatments at 45 and 50°C. E: control strain containing empty pJL43-RNAi. Error bars represent the standard deviation of three replicas from three independent experiments. The differences in conidial production of strains SFK19 and SFK38 were statistically undistinguishable (*p* < 0.05 using Student’s *t*-test) as compared to the wild-type and control strains. **(B)** Relative apical growth rates in Czapek minimal containing 0.45 M NaCl or 0.7 M KCl. Error bars represent the standard deviation of three replicas from three independent experiments. The symbol ^∗^ indicates that reductions in stress resistance of strains SFK19 and SFK38 were statistically significant (*p* < 0.05 using Student’s *t*-test) as compared to the wild-type strain. E: control strain containing empty pJL43-RNAi.

In *S. cerevisiae*, Sfk1 is important for response to heat shock (Audhya and Emr, 2002). For this reason, we tested thermal stress resistance of *P. roqueforti* strains. Remarkably, we did not found any difference in the ability to resist heat shock between the wild-type strain and transformants SFK19 and SFK38 (**Figure 5A**). On the other hand, we also tested hypertonic stress resistance, and we found interesting differences. Thus, the RNA-mediated silencing of *sfk1* decreased osmotic stress resistance to NaCl or KCl (**Figure 5B**). As average, the relative apical extension rate of transformants SFK19 and SFK38 was approximately 66% of wild-type levels in the presence of KCl or NaCl.

### The RNA-Mediated Gene-Silencing of *sfk1* Affects Cell Wall Integrity in *P. roqueforti*

**FIGURE 6:**
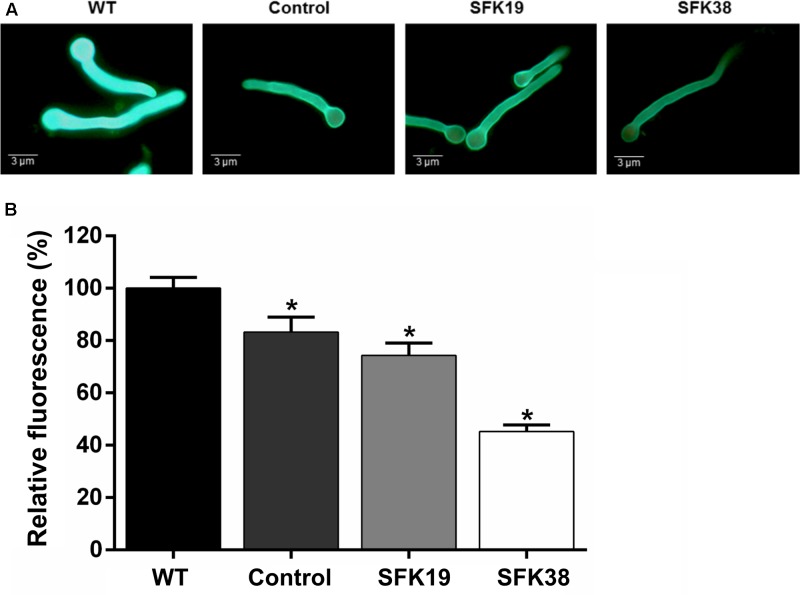
Effect of the *sfk1* gene on the cell wall integrity of *P. roqueforti* CECT 2905 (WT) and transformants SFK19 and SFK38. **(A)** Representative images of germinated conidia after calcofluor staining. Note the decreased fluorescence in transformants SFK19 and SFK38 as compared to the wild-type strain. As control of cell wall damage, *P. roqueforti* CECT 2905 was treated with “Lysing enzymes” from *T. harzianum*, as described in Section “Materials and Methods.” **(B)** Data were quantified and expressed as percentage of relative fluorescence, as described in Section “Materials and Methods.” The symbol ^∗^ indicates that reductions in intensity of fluorescence were statistically significant (*p* < 0.05 using Student’s *t*-test) as compared to the wild-type strain.

It has been suggested that Sfk1 could be important for cell wall integrity (see section “Discussion”), so we analyzed the effect of the RNA-mediated silencing of *sfk1* on the cell wall integrity in *P. roqueforti* by using calcofluor white (**Figure 6**). This compound is a fluorochrome molecule which binds to the chitin in the cell walls of the fungi. Therefore, a decrease in cell wall fluorescence intensity produced by calcofluor white is indicative of cell wall damage (Mendoza et al., 2016). Our results indicate that compared to the wild-type strain, transformants SFK19 and SFK38 have lower fluorescence intensity (**Figure 6A**). In the case of strain SFK19, it shows approximately 74% of the fluorescence of wild-type strain levels, whereas in SFK38 the effect is more dramatic, because this strain shows approximately 45% of the fluorescence of wild-type strain levels (**Figure 6B**). These data suggest that the knockdown of *sfk1* affected the cell wall integrity in transformants SFK19 and SFK38.

### The Knockdown of *sfk1* Decreases the Production of the Secondary Metabolites Roquefortine C, Mycophenolic Acid, and Andrastin A in *P. roqueforti*

**FIGURE 7:**
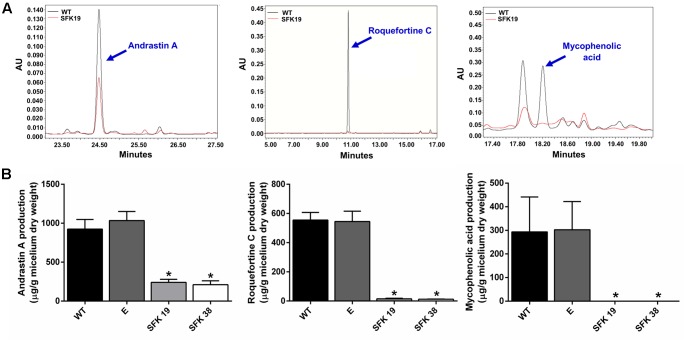
Production of secondary metabolites andrastin A, roquefortine C, and mycophenolic acid by *P. roqueforti* CECT 2905 and transformants SFK19 and SFK38. **(A)** Examples of HPLC trace chromatograms (300 nm) of the transformant SFK19 (red line) as compared to the wild-type strain (black line). In each panel, the peak representing the specific compound is indicated by a blue arrow. Note the reduction of the peaks in transformant SFK19 as compared to the wild-type strain. The same behavior was observed in transformant SFK30 (not shown). **(B)** Metabolites extracted were quantified and normalized by the dry weight of the fungal mycelia as described in Section “Materials and Methods.” Left panel: andrastin A. Center panel: roquefortine C. Right panel: mycophenolic acid. Error bars represent the standard deviation of three replicates in three independent experiments. Statistical analysis (Student’s *t*-test, *p* < 0.05) indicates significant differences between the production of the secondary metabolite by the wild-type strain and the transformants (^∗^). E: control strain containing empty pJL43-RNAi.

Roquefortine C, mycophenolic acid, and andrastin A are some of the main secondary metabolites produced by *P. roqueforti* (García-Estrada and Martín, 2016), so we studied if RNA-mediated silencing of *sfk1* affected their production. As shown in **Figure 7** the production of these secondary metabolites is depleted in transformants SFK19 and SFK38 compared to the wild-type strain. In the case of andrastin A, the wild-type strain produces approximately 900 μg/mg of the compound, whereas in the same conditions, the transformants SFK19 and SFK38 produce approximately 250 μg/mg (**Figure 7B**). More evident is the effect on roquefotine C. In this case, the wild-type strain produces approximately 560 μg/mg, whereas the transformants produce barely 15 μg/mg (**Figure 7B**). Finally, the most drastic effect was observed for mycophenolic acid. The wild-type strain produces approximately 300 μg/mg of this compound, but is almost undetectable in transformants SFK19 and SFK38 (**Figure 7B**). These results strongly suggest that the knockdown of *sfk1* decreases the production of the main secondary metabolites from *P. roqueforti.*

## Discussion

In *S. cerevisiae*, the overexpression of *sfk1* produces an interesting phenotypic change: different to the usual isotropic growth, yeasts overexpressing *sfk1* show filamentous-like growth phenotype (Jin et al., 2008). On the other hand, and by using overexpression and deletion mutants, it was demonstrated that *sfk1* has a positive effect in the thermal resistance in *S. cerevisiae* (Audhya and Emr, 2002). Further, the *sfk1* gene is overexpressed when *S. cerevisiae* is exposed to toxic substances such as benzene (North et al., 2011), trichothecenes (Suzuki and Iwahashi, 2012) and copper (Hodgins-Davis et al., 2012), suggesting that this gene could be related to the stress response in the yeast.

RNA-mediated silencing of *sfk1* reduced apical growth in *P. roqueforti* (**Figure 3**). This result matches well with those obtained in *S. cerevisiae*. In the yeast, the elimination of *sfk1* produced smaller colonies compared with the wild-type strain (Audhya and Emr, 2002), whereas the overexpression of the gene resulted in a filamentous-like growth (Jin et al., 2008). Although further work is necessary to extend the observations made in *P. roqueforti* to other filamentous fungi, our results and the previous results obtained in *S. cerevisiae* suggest that *sfk1* is a positive regulator of vegetative growth. In yeasts, it was demonstrated that Sfk1 stabilizes Stt4 at the plasma membrane (Audhya and Emr, 2002). Stt4 is a phosphatidylinositol 4-kinase involved in the production of phosphatidylinositol-4-phosphate (PI4P) and in turn, PI4P activates PKC pathway producing actin organization (Audhya and Emr, 2002). Accordingly, it can be hypothesized that the RNA-mediated silencing of *sfk1* in *P. roqueforti* resulted in an impaired organization of actin. Taking into account that actin has a pivotal role during polarized hyphal growth (Steinberg et al., 2017), the impaired organization of actin produced by the reduction of *sfk1* transcripts can explain the reduced apical growth observed in *P. roqueforti*. Further work is necessary to demonstrate this proposal.

According to our results, *sfk1* does not affect conidiation. In *P. roqueforti*, it has been proposed the existence of a dual control of conidiation by Gαi through the cAMP-dependent and cAMP-independent pathways (García-Rico et al., 2008a). In addition, recent evidence suggests that *pcz1*, probably through cAMP-independent pathway, stimulates conidiation in this fungus through positive regulation of the expression of conidiation-specific genes *brlA*, *wetA*, and *abaA* (Gil-Durán et al., 2015). Our results suggest that *sfk1* would not be involved in any of these pathways regulating conidiation.

Our results also indicate that *sfk1* has a negative role in conidial germination. Spore germination is triggered by environmental factors, which are sensed and transduced by signal transduction pathways that have been studied in detail in few species (Fillinger et al., 2002; Doehlemann et al., 2006; Krijgsheld et al., 2013). As in other fungi, *P. roqueforti* Gαi signaling has been demonstrated to control conidial germination (García-Rico et al., 2009) and recently it was suggested that *pcz1* may be participating in this Gαi-signaling pathway controlling conidial germination (Gil-Durán et al., 2015). Here we show for the first time that *sfk1* is also involved in the regulation of conidial germination. As deduced, the regulation of conidial germination in *P. roqueforti* is unclear, and more work is required to know how *sfk1* contributes to the control of spore germination process.

In *S. cerevisiae*, *sfk1* changes its expression pattern when the yeast is exposed to different toxic substances (North et al., 2011; Hodgins-Davis et al., 2012; Suzuki and Iwahashi, 2012), suggesting that this gene would be a general stress response determinant. Beyond this, its role in specific stressing conditions has not been fully evaluated and understood. Here, we found that the RNA-mediated silencing of *sfk1* reduced osmotic stress resistance in *P. roqueforti* (**Figure 5**). To the best of our knowledge, this is the first report describing the involvement of *sfk1* in osmotic stress response in fungi. Fungi sense osmotic stress through the HogA-MAPK pathway (Bahn et al., 2007; Hagiwara et al., 2016). Interestingly, a recent study has indirectly related HogA-MAPK pathway with Sfk1. Specifically, during their investigation about the role of Tor1 on hyphal elongation in *Candida albicans*, Su et al. (2013) found that: (i) Sfk1 may be a Tor1 regulator or perform a function in the Tor1 pathway; (ii) Tor1 signaling downregulates Hog1 basal activity. Accordingly, and assuming that these regulators are likely conserved in *P. roqueforti*, it can be hypothesized that the RNA-mediated silencing of *sfk1* reduced osmotic stress resistance in *P. roqueforti* through the HogA-MAPK pathway. Further studies are needed to demonstrate this interesting hypothesis.

Audhya and Emr (2002) demonstrated that Sfk1 is a positive regulator of thermal stress resistance in *S. cerevisiae*. However, we did not found differences in thermal stress resistance between *P. roqueforti* wild-type and the SFK19 and SFK38 transformants. Mechanisms of thermal stress resistance in fungi are multifactorial and not fully understood. Several reports have showed that fungi regulate thermal stress response through the same HogA-MAPK pathway described above (Alonso-Monge et al., 2009; Ji et al., 2012). Further, as specific responses to elevated temperature, fungi modify membrane composition and produce intracellular molecules that act as thermal protectants, such as heat shock proteins and trehalose (Leach and Cowen, 2014; Hagiwara et al., 2016). Our results suggest that different to *S. cerevisiae*, *sfk1* would not influence on these response mechanisms in *P. roqueforti*, so the thermal stress resistance pathway in *P. roqueforti* could be somewhat different from yeast.

In *S. cerevisiae*, Stt4 is required for cell wall integrity (Audhya et al., 2000). In turn, Stt4 is tethered to the plasma membrane by Sfk1 (Voelker, 2005). These evidences suggest that Sfk1, indirectly, could be important in maintenance of cell wall integrity (Audhya and Emr, 2002; Levin, 2005). Our results support this prediction, because the RNA-mediated silencing of *sfk1* reduced cell wall integrity in *P. roqueforti* (**Figure 6**). However, how exactly *sfk1* affects cell wall integrity in fungi is unknown. To the best of our knowledge, at least two putative mechanisms have been proposed: (i) the direct recruitment/activation of effector proteins by PI4P (generated by Stt4) and the downstream metabolite phosphatidylinositol-4,5-biphosphate; (ii) the activation of effectors through the MAPK- cell wall integrity (CWI) pathway (Audhya and Emr, 2002; Levin, 2005).

The RNA-mediated silencing of *sfk1* drastically reduced the production of andrastin A, roquefortine C and mycophenolic acid in *P. roqueforti* (**Figure 7**). Although the enzymatic pathways for the biosynthesis of these secondary metabolites in *P. roqueforti* have been recently elucidated (Kosalková et al., 2015; Del-Cid et al., 2016; Rojas-Aedo et al., 2017), the cellular mechanisms controlling their biosynthesis are not fully understood. As example, none of the cluster for the biosynthesis of these compounds contains a gene with similarity to a putative transcription factor (Kosalková et al., 2015; Del-Cid et al., 2016; Rojas-Aedo et al., 2017), suggesting that their biosynthesis would be controlled by global regulators. In the case of roquefortine C, studies performed in different *Penicillium* species (*P. roqueforti*, *P. chrysogenum*, and *P. decumbens*) indicate that the production of this metabolite could be controlled by GαI protein and the regulator of conidiation BrlA (García-Rico et al., 2008b, 2009; Qin et al., 2013) but remarkably, its biosynthesis would not depend on LaeA (Kosalková et al., 2009), a global regulator that controls secondary metabolism in several fungi. Regarding mycophenolic acid and andrastin A, to the best of our knowledge regulators controlling their biosynthesis have not been described yet. Therefore, our finding that *sfk1* regulates the biosynthesis of roquefortine C, mycophenolic acid and andrastin A is an important step forward in understanding the processes that regulate the biosynthesis of secondary metabolites in *P. roqueforti*.

## Author Contributions

IV, RG-R, MC, LM, GL, and RC conceived and designed the experiments, contributed reagents/materials, analyzed the data and supervised work. CT, CG-D, JR-A, EM, IV, and PC carried out the experiments and analyzed the data. EM and RC performed bioinformatics analysis. IV and RC drafted the manuscript. All authors have read and approved the manuscript.

## Conflict of Interest Statement

The authors declare that the research was conducted in the absence of any commercial or financial relationships that could be construed as a potential conflict of interest.
